# Editorial: Plant genotyping: from traditional markers to modern technologies, volume II

**DOI:** 10.3389/fpls.2025.1755453

**Published:** 2025-12-16

**Authors:** Yuri Shavrukov, Sunita Gupta, Gulmira Khassanova

**Affiliations:** 1 College of Science and Engineering (Biological Sciences), Flinders University, Adelaide, SA, Australia; 2 Rajasthan Agricultural Research Institute, Sri Karan Narendra (SKN) Agriculture University, Jaipur, India; 3 Institute of Agriculture and Forestry, S. Seifullin Kazakh AgroTechnical Research University, Astana, Kazakhstan

**Keywords:** genetic polymorphism, molecular markers, molecular plant breeding, plant genotyping, simple sequence repeat (SSR), single nucleotide polymorphism (SNP)

The Research Topic, with 15 accepted and published papers, confirms the importance of research focused on plant genotyping. These studies cover various methods, ranging from traditional simple sequence repeats (SSRs) to single nucleotide polymorphisms (SNPs), based on advanced and modern technologies. The published articles address various questions from evolution and phylogeography to the sequencing of entire genomes and the development of molecular markers and their application in practical breeding. We summarise our Research Topic below.

Molecular markers are very important components of plant genotyping and functional markers (FMs) are powerful tools in plant breeding, as described in the review paper in our Research Topic. The authors detailed the discovery, development, and verification of FMs using forward and reverse genetics approaches for gene identification and quantitative trait polymorphism analyses. The practical application of FMs in marker-assisted selection (MAS) and backcrossing was illustrated with many examples from various crops. MAS is advantageous for selecting simple traits. However, for complex traits, genomic selection based on whole genome information has been reported to be more effective. The authors concluded that FMs can serve as key tools in breeding efforts to address diverse environmental and agricultural challenges (Park et al.).

The loop-mediated isothermal amplification (LAMP) method was successfully optimised for the genotyping of the plant species *Rhodiola crenulata* (Hook.f. & Thomson) H.Ohba, an important resource in traditional Chinese medicine. This method is based on identified SNPs that were then confirmed in various genotypes of accessions and their hybrids using the classical cleaved amplified polymorphic sequence (CAPS) method. The authors developed the LAMP method using a qPCR instrument with the incorporation of two colour indicators. The accuracy of the presented genotyping results is evident, as demonstrated by perfect identifications and designations between closely related *Rhodiola* species. The presented results also clearly differentiated *Rh. crenulata* from other species and their hybrids, which is crucially important for downstream applications in the pharmaceutical industry (Hao et al.).

Classic genotyping with SSR markers was used to study the genetic polymorphism and diversity of 177 avocado trees in La Palma, in the Canary Islands. This is a very elegant publication that used only nine selected SSR markers to demonstrate effective fingerprinting of avocado genotypes. With only a small number of SSR markers, this provides a valuable tool due to its simple and cheap analysis using the reference data available. The authors reported significant genetic polymorphism and diversity within avocado genotypes from the Canary Islands. They concluded that the conservation of this germplasm should be a priority to ensure its availability for future avocado agricultural innovation and improvement efforts (Perez et al.).

*Salicornia*, a salinity tolerant horticultural crop, is a new plant species in Europe, with taxonomic studies limited by its unique morphology, broad phenotypic plasticity, and few distinguishing characteristics. The authors used various methods to identify genetic polymorphism and study SSR fingerprinting, including nuclear DNA analysis, marker-based DNA barcoding, the application of diagnostic SNPs within the external transcribed spacer and three promising SSR markers, and finally, morphological traits of plant habits and flowers for accurate identification of *Salicornia* species. The authors concluded that differentiation among species is crucial for initiating breeding programmes, particularly given previous morphological misidentification (Fussy et al.).

In the deciduous shrub plum-leaved holly, *Ilex asprella* (Hook. et Arn.) Champ. ex Benth, a common Chinese herb, six types of SSRs with 137,443 loci in total were identified across the entire genome. This enormous piece of research developed a fluorescence-based identification method for SSR markers and contains excellent plant illustrations. Ultimately, 15 highly polymorphic primers were selected to study genetic polymorphism in *I. asprella* species. The authors reported significant differences in genetic diversity among the three identified populations. It was concluded that gene exchange between populations can be used as an important guideline for further selection based on SSR genetic polymorphism and for the commercial breeding of new varieties for the pharmaceutical industry (Li et al.).

Plant genotyping was used in the study of phylogeography in *Morinda officinalis* How, a Chinese native tropical and subtropical plant species from the coffee family Rubiaceae that is important in traditional medicine. The authors reported on the genealogy and simulated the distribution of *M. officinalis* plants in Chinese regions based on Bayesian phylogenetic analyses of chloroplast and nuclear gene sequences. The genotyping results indicated that the expansion of the species occurred during the Last Interglacial period. Molecular phylogenetic evidence was provided for the origin of *M. officinalis*, indicating that the species adapted to a timeline of major geological and climatic episodes rather than local events (Zhang et al.).

Genetic polymorphism was studied in orchardgrass (*Dactylis glomerata* L.), an important perennial and cool-season forage crop, using diversity arrays technology (DArT-seq), which is based on next generation sequencing (NGS) platforms. In this report, 91 accessions from 27 geographic locations, primarily from Türkiye, were genotyped for subsequent genome-wide association study (GWAS) analysis of biomass production. The presented results, based on 2,913 high-quality DArT markers, showed a distinct distribution into three clusters. The authors reported very strong associations between genotypes in clusters and their dry biomass production. Four DArT markers associated with biomass production were identified, which is important for future breeding programmes (Tanveer Altaf et al.).

Similar genotyping technology, DArT-seq, was used for a GWAS analysis of soybean yield and yield-related traits under drought conditions. After filtration, 16K DArT markers were applied to 183 soybean cultivars grown in non-irrigated and well-watered field trials. The authors reported on 41 quantitative trait nucleotides (QTNs) that were identified as significantly associated with nine studied yield-related traits. The presented results revealed the 10 most significant QTNs and eight most promising putative candidate genes, which were tested for gene expression using RT-qPCR under drought conditions. The authors concluded that the identified and verified genes can be used in MAS to develop novel, drought tolerant soybean cultivars (Amangeldiyeva et al.).

A genotyping-by-sequencing method based on the Illumina NovaSeq X system was applied to 61 accessions of traditional Italian landraces of potato (*Solanum tuberosum*) to study their genetic polymorphism and agricultural homogenisation. The reported results indicated clear genetic differentiation of the Italian landraces into three molecular clusters, with minimal overlap with commercial cultivars. This confirms their unique genetic identity, which is very distant from a reference panel of potato genotypes. The authors concluded that landraces represent an important genetic diversity of traits that contribute to the development of resilient and productive potato cultivars (Martina et al.).

Comprehensive whole genome sequencing (WGS) was used, based on the Illumina NovaSeq 6000 platform, to study seed coat colour in four composite populations of common bean (*Phaseolus vulgaris* L.). WGS identified 8.6 million SNPs, and 118 significant genetic regions were associated with seed coat colour. The authors reported candidate genes related to phosphatidylinositol bisphosphate binding, exocytosis, and vesicle-mediated transport, resulting in pigment deposition in the seed coat. They concluded that variation in seed coat colour is controlled by both regulatory and structural genes, an important starting point for further breeding programmes (Plestenjak et al.).

Data reports are a very different type of article, but vitally important for sharing information on the large amount of data generated when analysing plant species and their genotyping. Four data reports were published in our Research Topic. WGS technology was applied to 56 accessions of the woody oil tree, sea buckthorn (*Hippophae rhamnoides* L.), to study different fruit traits, providing data for the development of genetic technologies and further breeding. WGS with Illumina reads was performed to a depth of 25× genome coverage, yielding data on approximately 4 million DNA polymorphisms. The genotypes were distributed among five clusters in a molecular phylogenetic tree, which is a useful tool for plant breeding (Melnikova et al.). A similar WGS strategy was applied to study 23 *Populus* accessions from Russia, representing 11 different taxa. This was an important step in producing a complete picture of the genetic diversity within the taxon and in comparing it with poplars from other countries, including 74 genotypes available in the NCBI database. The seven clades identified in the molecular dendrogram accurately demonstrated a similarity among the studied *Populus* genotypes (Borkhert et al.).

The chloroplast genome was studied in two data reports. Two species in the *Neolamarckia* genus, *N. cadamba* and *N. macrophylla* (Roxb.) Bosser, from the Rubiaceae family, commonly known as white and red jabon, respectively, are fast-growing native trees in Indonesia, and widely used in industrial plantations for forest and land rehabilitation. Data obtained from the WGS of *Neolamarckia* species were used to study the chloroplast genome and screen SSR markers (Dwiyanti et al.). *Ardisia silvestris*, from the Primulaceae family, is a potential medicinal plant species native to subtropical and tropical areas in Vietnam and China. Using the Illumina sequencing platform, the complete chloroplast genome of *A. silvestris* was studied in accessions from Vietnam, providing the initial chloroplast genome data for further phylogenetic study and molecular marker development (Nguyen et al.).

A systematic review on the flat peach, *Prunus persica* (L.) Batsch provided an analysis of 40 publications and indicated that peach fruit shape is associated with a very large inversion in chromosome 6, a structural variation in the plant genome. The authors concluded that flat peaches are gaining recognition as a distinct and valuable crop with high fruit quality (Gedamu et al.).

In conclusion, plant genotyping will always be a popular topic since it is an important and unavoidable requirement in plant biology research. Regardless of the technology used, whether traditional or modern, which is constantly developing, all plant biologists must understand and incorporate plant genotyping approaches into their research for a clear and accurate assessment of genetic polymorphism in the plant material forming their area of focus.

